# A Pilot Randomized Controlled Trial Investigating MBSR for Parkinson’s Disease Patients and Their Caregiving Partners: Effects on Distress, Social support, Cortisol, and Inflammation

**DOI:** 10.1007/s12671-022-01874-y

**Published:** 2022-06-03

**Authors:** Chelsea J. Siwik, Kala Phillips, Irene Litvan, Paul Salmon, Allison Rodgers, Megan Jablonski, Sandra E. Sephton

**Affiliations:** 1 Department of Psychological and Brain Sciences, University of Louisville, 2301 S 3rd St., Louisville, KY 40292, USA; 2 Osher Center for Integrative Medicine, University of California, San Francisco, 1545 Divisadero St., San Francisco, CA 9411, USA; 3 Rehabilitation Medicine, University of Washington, 908 Jefferson St, Seattle, WA 98104, USA; 4 Parkinson and Other Movement Disorder Center, Department of Neurosciences, University of California, San Diego, 9500 Gilman Drive, La Jolla, CA 92093, USA; 5 Richard L. Roudebush VA Medical Center, 1481 W 10th St, Indianapolis, IN 46202, USA; 6 Frazier Rehabilitation Institute, University of Louisville, 20 Abraham Flexner Way, Louisville, KY 40202, USA; 7 James Graham Brown Cancer Center, University of Louisville, 29 S Jackson St, Louisville, KY 40202, USA; 8 Department of Psychology, Brigham Young University, 1001 KMBL, Provo, UT 84602, USA

**Keywords:** MBSR, Parkinson’s disease, Caregiver, Patient-partner dyads, Inflammation, Cortisol

## Abstract

**Objectives:**

To examine the preliminary effects of the mindfulness-based stress reduction (MBSR) program in the management of biopsychosocial stress–related changes associated with Parkinson’s disease (PD) among patient/caregiving-partner dyads.

**Methods:**

PD patient/caregiving-partner dyads (*N* = 18) early in the disease trajectory were recruited from a university-affiliated movement disorders clinic and were randomized (1:1) to either the MBSR intervention or the control condition (treatment as usual [TAU]). Mixed methods ANOVAs were conducted to examine primary outcomes (disease-specific distress, perceived social support, circadian rhythmicity [cortisol], and markers of inflammation [IL-6, TNF-alpha, IL-1beta]) between groups (MBSR vs. TAU) among patients and caregiving partners separately.

**Results:**

No participants were lost to follow-up. Given the pilot nature of the current investigation, findings should be interpreted as exploratory opposed to confirmatory. Following MBSR, PD patients reported an increase in disease-specific distress and intrusive thoughts and demonstrated a decrease in mean bedtime cortisol and IL-1beta from baseline to follow-up compared to TAU. Caregiving partners who received MBSR reported an increase in perceived social support and demonstrated improved rhythmicity of diurnal cortisol slopes from baseline to follow-up compared to TAU.

**Conclusions:**

Both patients and caregiving partners who received MBSR demonstrated improvements in biomarkers of circadian function, and patients evidenced a decrease in a biomarker of systemic inflammation, pointing to an important area of further investigation. Given that patients reported an increase in disease-specific distress and intrusive thoughts, the salutary effects of MBSR may be experienced physiologically prior to, or in lieu of, psychological effects, although this should be explored further, especially given the improvement in perceived social support reported by caregiving partners.

Parkinson’s disease (PD) is a progressive neurodegenerative movement disorder characterized by neurodegeneration of dopamine-producing neurons in the substantia nigra, an area in the brain involved in controlled movement and coordination (Hughes et al., 1992) and other non-motor pathways. Although PD is most commonly recognized by motor symptoms, defined as bradykinesia plus resting tremor or rigidity (Postuma et al., 2018), there are a number of non-motor changes that accompany PD, including disease-related distress, depression, anxiety, sleep disruption, including rapid eye movement (REM) sleep behavior disorder, and pain, which can lead to poorer disease outcomes and quality of life (Broen et al., 2016; Chaudhuri et al., 2006). Due to the progressive nature of the disease, common stressors are anticipation of worsening symptoms, worry surrounding the rate at which degeneration will occur, and fear of loss of independence, death, and becoming a burden to family. Patients with PD have reported mental and psychosocial symptoms to be more severe and distressing than problems related to motor-related difficulties (Abudi et al., 1997; Backer, 2000; Habermann, 1996). Despite the prevalence of these non-motor changes and known sources of disease-related distress, PD treatment tends to focus exclusively on motor symptoms, highlighting a gap in care.

In addition to the neurologic changes that are characteristic of PD, a growing number of studies have evidenced poorer physiological and immune function among patients with PD, including elevated mean cortisol levels (Breen et al., 2014), flattened diurnal cortisol slopes (Hartmann et al., 1997), and elevated proinflammatory cytokines, including IL-6, IL-1beta, TNF-alpha, and CRP (Reale et al., 2009; Song et al., 2009). Emerging evidence suggests disrupted circadian rhythmicity may not only be linked to non-motor symptoms of PD, such as poor sleep (Breen et al., 2014), but may be integral to the pathology of the disease and may even accelerate the disease (Willison et al., 2013). Similarly, elevated levels of proinflammatory markers have been linked to more severe instances of non-motor symptoms, including depression (Lindqvist et al., 2013), and higher proinflammatory/lower anti-inflammatory markers have been shown to predict more rapid progression of motor symptoms (Williams-Gray et al., 2016). It remains unclear whether these biological shifts are attributable to the disease itself, the downstream consequences of psychological reactions to the disease, or a combination of both. Regardless, non-pharmacologic interventions that improve circadian and immune function without introducing pharmacologic side effects may reduce motor and non-motor symptoms among those with PD.

Given the well-established beneficial effects of social support in the context of medical illness (Wang et al., 2003), it is no surprise that social support has been shown to ameliorate the effects of disease among individuals with PD as well (Simpson et al., 2006). Defined in terms of emotional, instrumental, and/or informational assistance (Finfgeld-Connett, 2005), an individual’s spouse, partner, children, family, friends, colleagues, neighbors, and/or community members can provide social support. However, relationships can be challenging to maintain as the disease progresses, and greater dissatisfaction with social support has been linked to greater psychological distress (Simpson et al., 2006). Thus, there may be an opportunity to improve a PD patient’s overall health by buffering against the perceived decline in social support that tends to accompany disease progression.

It is common for partners of individuals with PD to adopt the role of informal caregiving. Results from a Canadian public health survey conducted in 2010–2011 indicated 84% of individuals with PD who reported receiving assistance relied at least in part on informal caregivers (e.g., family, friends, neighbors), and 56% relied solely on assistance from informal caregivers (Wong et al., 2014). As such, caregivers similarly experience shifts in their social role as well as the anticipation of inevitable changes in their partner’s physical, cognitive, emotional, and interpersonal functioning. Caregiving partners are confronted with the burden of two roles: as partners, they witness significant changes in their partner’s functioning, often an emotionally taxing experience, and as caregivers, their role becomes increasingly demanding as symptoms progress and the individual becomes more disabled. These stressors can translate to caregiving burden (Karlstedt et al., 2020), fatigue, and difficulty with adjustment (Martinez-Martin et al., 2005), potentially leading to a decline in the caregiver’s health. One study reported that over 40% of informal PD caregivers indicated their health had suffered as a result of their caregiving role and 27% felt caring had made them physically ill (Schrag et al., 2006). Half of these caregivers demonstrated increases in depression and two-thirds indicated a decline in social activities, which is noteworthy given that perceived social support is protective against caregiver burden (Edwards & Scheetz, 2002). Declines in the caregiver’s health could in turn worsen the patient’s health and well-being. In one study, caregiver burden correlated with patients’ depression and quality of life (Schrag et al., 2006), and patients’ non-motor symptoms have demonstrated a greater contribution to caregiving burden than motor symptoms (Mosley et al., 2017). These findings indicate that the presence and decline in non-motor symptoms appear to be shared within the patient/caregiver dyad, yet limited attention is currently given to the needs of caregiving partners.

Given the high rates of stress reported by caregiving partners (Schrag et al., 2006), it is reasonable to theorize they too are experiencing dampened physiological and poorer immune functioning. It is well documented that chronic stress is linked to poorer hypothalamic–pituitary–adrenal (HPA) function, meaning higher diurnal mean cortisol and flatter diurnal cortisol slope (Miller et al., 2007) as well as poorer immune function (Dhabhar, 2014). Yet these biomarkers have not been examined among caregiving partners of individuals with PD. Exploration into these markers of health could elucidate caregivers’ reports of a decline in health (Schrag et al., 2006) as well as caregiving as a risk factor for mortality (Schulz & Beach, 1999).

Taken all together, a diagnosis of PD is stressful and evokes biopsychosocial changes for both the patient and the caregiving partner. Although individuals with PD experience stress-related physiological shifts that could predispose for poorer health and quality of life, current treatments tend to focus exclusively on the management of the patient’s motor systems. As such, a more comprehensive approach to the management of non-motor symptoms for both patients and their caregiving partners is currently lacking. To investigate a more comprehensive approach, the current study aimed to examine the preliminary effects of a mindfulness-based stress reduction (MBSR) program among patient/caregiving partner dyads in the management of non-motor, stress-related changes associated with PD.

MBSR is a widely known clinical stress reduction program established by Kabat-Zinn (1982) that teaches mindfulness principles and practices through awareness exercises, body scan, sitting meditation, and hatha yoga. MBSR was designed for chronically ill patients and has since demonstrated both psychological and physiological benefits across many clinical populations (Bohlmeijer et al., 2010; Ledesma & Kumano, 2009), making it a promising approach for those afflicted by PD. The 8-week program is composed of 150-min, weekly group sessions with a recommendation for 45 min of daily at-home practice to enhance attitudinal foci (e.g., the beginner’s mind, non-judgment, patience, trust, non-striving, letting go, acceptance). Given the future-oriented nature underlying the stressors that accompany PD, it is plausible to hypothesize a present-moment-focused intervention may be particularly beneficial for patients and their caregiving partners.

Indeed, a handful of studies have examined mindfulness in the context of PD. A pilot study conducted by Cash et al. (2016) demonstrated an increase in mindfulness and language-functioning and a decrease in symptoms of depression following MBSR among PD patients and their caregivers/family members. However, dyadic participation was not required in this study, meaning only ten participants (*N* = 39) were in a caregiving role, limiting conclusions about the efficacy of MBSR for simultaneous management of dyadic patient/caregiver stress. Although preliminary, this study points to MBSR as feasible and efficacious for improving cognitive and emotional functioning among this population, which is consistent with findings from a different study that demonstrated increases in graymatter density in key brain regions (e.g., right amygdala and bilaterally in the hippocampus) in PD patients after a mindfulness-based intervention (Pickut et al., 2013). Importantly, a recent study demonstrated PD patients and caregiving partners who possess greater dispositional mindfulness demonstrate less stress and depressive symptoms and greater social support, sleep quality, and health-related quality of life (Hicks et al., 2019), further evidencing mindfulness as an appropriate intervention for coping with stress-related symptoms among this population. While these studies build a basis for evidencing the benefits of mindfulness among PD patients and caregiving partners, no research to date has comprehensively examined the effects of MBSR across biopsychosocial markers of stress among PD patient/caregiving partner dyads.

The current investigation was a pilot RCT designed to explore the preliminary effects of MBSR among PD patient/caregiving-partner dyads in the management of non-motor, stress-related changes associated with PD. Adapted from the published model of stress-health relationships (Salmon et al. (2011), the present study investigated preliminary changes in four biopsychosocial markers of health following MBSR, as dispositional mindfulness has been shown to be a protective factor against psychosocial stress outcomes among PD patient/caregiving-partner dyads (Hicks et al., 2019). We hypothesized MBSR would decrease appraisal of disease-specific distress, increase perceived social support, improve HPA-axis function (e.g., lower mean cortisol, steeper diurnal cortisol slope), and improve immune function (decrease proinflammatory cytokines) among both PD patients and their caregiving partners.

## Methods

### Participants

PD patient/caregiving-partner dyads were recruited from a Movement Disorders Clinic specializing in PD at a University-affiliated Rehabilitation Institute. Patients were referred by collaborating physicians and/or medical records were screened using patient eligibility criteria. Inclusion criteria for patient participants consisted of (1) a diagnosis of idiopathic PD within 3 years based on the UK PD Brain Bank criteria (Gibb & Lees, 1988); (2) stage II or III according to the Hoehn and Yahr Parkinson’s Disease Severity Scale (Hoehn & Yahr, 1998); (3) adequate cognitive functioning defined as a score ≥ 25 on the Mini-Mental Status Examination (MMSE; Folstein et al. (1983); (4) ≥ 40 years of age; and (5) proficiency in written and spoken English. Eligible caregiving partners identified as the “primary caregiver” for the patient were required to provide ≥ 4 h of care per day, demonstrate adequate cognitive functioning (MMSE score ≥ 28), and be proficient in written and spoken English. Exclusion criteria for both patients and caregiving partners included a serious medical or psychological illness that would preclude participation and use of systemic hydrocortisone-based steroids that could interfere with accurate salivary cortisol assessment.

Eligible dyads were identified by systematic chart review and were introduced to study personnel by collaborating physicians during a medical appointment at the clinic. Study personnel provided information about the study. Of the 141 patients screened over the course of approximately 6 months, 86 were determined eligible, and 19 eligible and interested dyads enrolled in the IRB-approved study. One dyad withdrew shortly after consenting due to scheduling conflicts with study activities. The final sample consisted of eighteen dyads (*N* = 36).

Participants reported on clinical and demographic characteristics and factors known to affect cortisol secretion (e.g., medication, alcohol, tobacco, caffeine). See Table 1 for a description of sample demographics.

### Procedures

After eligible dyads were introduced to study personnel, trained graduate students conducted a brief mental status evaluation to confirm adequate cognitive functioning. Study physicians re-assessed PD staging criteria. Eligible patients and caregiving-partner dyads jointly attended two baseline assessment sessions held at a university laboratory. During the first session, each participant provided informed consent. Patients completed two physical movement tests, the Berg Balance Scale (Qutubuddin et al., 2005) and the Get up and Go Test (Morris et al., 2001), to ensure their ability to participate in the yoga portion of MBSR. Partners completed a brief interview on their caregiving experience. Both patients and caregiving partners were provided home-based data collection materials that included instructions, questionnaires assessing factors that could influence cortisol sampling, and a salivary cortisol collection kit containing salivettes. Participants were asked to collect three saliva samples per day for three consecutive days (a total of nine samples) immediately upon waking (prior to getting out of bed), 30 min after waking, and at bedtime. Participants were instructed not to put anything in their mouths 30 min prior to collecting a sample and to store samples in the refrigerator.

During the second session in the laboratory, patient/caregiving-partner dyads returned completed at-home data collection materials and provided a blood sample for immune marker assessment. Patient/caregiving-partner dyads were then blindly and randomly assigned (1:1) to an 8-week MBSR intervention or treatment as usual (TAU) by study personnel who did not have contact with participants. Dyads randomly assigned to the control condition received usual medical care and adjunct treatment recommended by their physician (e.g., physical therapy).

Dyads randomly selected for the MBSR intervention completed the program at an off-site location unaffiliated with the hospital clinic. The intervention was led by a faculty licensed clinical psychologist with MBSR training from the UMASS Center for Mindfulness. The present MBSR intervention consisted of eight, 2-h, weekly sessions, and a day-long meditation retreat (see Table 2). Participants were encouraged to complete home-based meditation practice for 30–45 min per day, 6 days per week, individually or with their caregiving partner. They were provided a workbook, pre-recorded meditation tracks, and a log to track their mindfulness practice.

All participants regardless of condition (MBSR or TAU) returned to the laboratory within 1 week of the conclusion of the intervention for follow-up assessment. Follow-up data collection mirrored baseline data collection procedures (i.e., an initial visit, at-home data collection, and a subsequent visit to return materials and conduct a blood draw).

### Measures

#### Disease-Specific Distress

The Impact of Events Scale (IES) is a 15-item self-report measure assessing the impact of events perceived as distressing (Horowitz et al., 1979). When responding to items on the measure, patients and caregiving partners were asked to consider the impact of the PD diagnosis and treatment. A total score was calculated by summing all items. Two subscales, intrusion and avoidance, were calculated by taking the average of 8 items. Greater scores indicate greater levels of disease-specific distress. Among the combined sample of PD patients and caregivers, the IES demonstrated strong reliability at baseline and follow-up, respectively (Cronbach’s *α* = 0.92, 0.89; McDonald’s *ω* = 0.92, 0.88).

#### Social Support

The Interpersonal Support Evaluation List (ISEL) is a 40-item self-report measure designed to assess the perceived availability of social report and overall functional support (Cohen & Hoberman, 1983). It contains four 12-item subscales: tangible, appraisal, self-esteem, and belonging. Caregiving partners were instructed to indicate how frequently they felt the items were true regarding their partner’s diagnosis and treatment of PD. Greater scores on the ISEL suggest a greater degree of perceived social support. Among the combined sample of PD patients and caregiving partners, the ISEL demonstrated strong reliability at both baseline and follow-up, respectively (Cronbach’s *α* = 0.93, 0.95; McDonald’s *ω* = 0.92, 0.94).

#### Diurnal Salivary Cortisol

Each participant collected a total of nine cortisol samples at home for three consecutive days at waking, 30 min after waking, and bedtime. Once returned to the laboratory, samples were centrifuged, aliquoted, and frozen at − 80 °C until assay. Cortisol levels were assessed using a luminescence immunoassay (IBL International, Hamburg, Germany). Assay sensitivity was 0.003 ug/dL. Inter-assay CV was 8.98% using the low control and 5.95% using the high control. Intra-assay CV was 10.2% for the low control and 7.2% for the high control. Raw cortisol values were log-transformed to correct for skew, from which three parameters were calculated: overall mean cortisol, mean bedtime cortisol, and diurnal cortisol slope. Overall mean cortisol was calculated from all sample times across the 3 days and mean bedtime cortisol was calculated from all bedtime samples across 3 days. Diurnal cortisol slope was calculated excluding post-waking samples using the unstandardized beta of natural log-transformed cortisol regressed on collection time (Kraemer et al., 2006). Samples collected less than 30 min or more than 60 min after waking for the “post-wake” sample were excluded (*n* = 4).

#### Serum Proinflammatory Cytokines

Two blood samples were collected per participant at both baseline and follow-up. One tube was centrifuged (spun at 1300 RCF for 10 min and 25 °C), aliquoted, and frozen at − 80 °C within 2 h of blood draw. Serum was assayed using R&D Systems EIA to quantify IL-6. The intra-assay and inter-assay CVs for the high control were 4.54% and 5.74%, respectively, for IL-6. The second tube of heparinized whole blood was incubated for 24 h with phytohemagglutinin (PHA), a T-cell mitogen and initiator of mitotic activity, under a sterile biological hood. The media plate that was used was removed from the freezer and thawed for 45–60 min in the CO_2_ incubator prior to use. The stimulated samples were microcentrifuged for 5 min at 12,000 rpm and 4 °C, aliquoted, frozen at − 80 °C, and assayed using R&D Systems EIA for TNF-alpha and IL-1beta. Because results were too low to read with the standard curve, assays were diluted. The diluted intra-assay and inter-assay CVs for the high control were 10.35% and 7.58% for TNF-alpha and 3.99% and 5.24% for IL-1beta, respectively. Values that fell > 4 standard deviations from the mean were considered outliers and excluded (IL-6: *n* = 1, IL-1beta: *n* = 3). Two samples were excluded due to limited sample.

### Data Analyses

Analyses were conducted in Statistical Package for the Social Sciences (IBM SPSS version 27.0). Using the splitfile function to separate patients from caregiving partners, two-way mixed ANOVAs were conducted to compare pre-to-post changes across conditions (MBSR versus TAU). A randomization check was conducted using independent sample *t*-tests comparing patients and caregiving partners separately across groups on outcome measures at baseline.

## Results

Patients were early in disease progression and did not report significant motor functioning impairment on the Hoehn and Yahr (1998) scale (*M* = 1.88, *SD* = 0.31) or the United Parkinson’s Disease Rating Scale (*M* = 16.7, *SD* = 11.6). Only one PD patient endorsed requiring assistance for independent movement. Most dyads (17/18) were romantic partners (married or heterosexual couples) and the remaining dyad identified as same-gender friends.

The randomization check indicated no significant differences in primary outcomes between participants randomized to MBSR compared to TAU at study entry. Results are summarized in Table 3. No participants were lost to follow-up. Participants in the MBSR group completed a mean of 2028 min (*SD* = 939) of at-home practice, which is equivalent to 36 min per day, and attended an average of 8 classes (*SD* = 1).

Due to the pilot nature of the current investigation and limited sample size, results should be interpreted cautiously. Among patients, there was a significant interaction between intervention and disease-specific distress, *F*(1, 15) = 5.61, *p* < 0.05, partial *η*^2^ = 0.27. Contrary to our hypothesis, disease-specific distress increased slightly among patients in the MBSR group (pre: *M* = 12.3, *SD* = 14.6; post: *M* = 13.2, *SD* = 11.2), whereas disease-specific distress decreased for patients who received TAU (pre: *M* = 16.3, *SD* = 16.9; post: *M* = 8.9, *SD* = 9.9). There was also a significant interaction between the intervention and intrusive thoughts, *F*(1, 15) = 7.51, *p* < 0.05, partial *η*^2^ = 0.33, as measured via the intrusion subscale of the IES. Patients in the MBSR group had a significant increase in intrusive thoughts (pre: *M* = 3.3, *SD* = 5.1; post: *M* = 4.4, *SD* = 4.1), whereas patients who received TAU reported a decrease in intrusive thoughts (pre: *M* = 7.0, *SD* = 8.2; post: *M* = 2.4, *SD* = 3.1). There was a significant interaction between intervention and log-transformed bedtime mean cortisol, *F*(1, 15) = 6.60, *p* < 0.05, partial *η*^2^ = 0.31. Patients in MBSR demonstrated a greater decrease in bedtime cortisol (pre: *M* = − 2.4, *SD* = 0.55; post: *M* = − 3.12, *SD* = 0.56) than patients who received TAU (pre: *M* = − 2.7, *SD* = 0.70; post: *M* = − 2.85, *SD* = 0.74). There a significant interaction between intervention and PHA-stimulated IL-1beta, *F*(1, 13) = 5.08, *p* < 0.05, partial *η*^2^ = 0.28. Patients in MBSR demonstrated a decrease in PHA-stimulated IL-1beta (pre: *M* = 1.57, *SD* = 1.15; post: *M* = 1.06, *SD* = 0.71), whereas patients who received TAU had a large increase in IL-1beta (pre: *M* = 1.24, *SD* = 1.29; post: *M* = 3.90, *SD* = 3.71).

Among caregiving partners, there was a significant interaction between intervention and perceived social support, *F*(1, 16) = 10.85, *p* < 0.01, partial *η*^2^ = 0.40. Caregiving partners who received MBSR reported an increase in perceived social support (pre: *M* = 25.9, *SD* = 5.5; post: *M* = 27.1, *SD* = 4.7), whereas those who received TAU reported a decrease in perceived social support (pre: *M* = 27.1, *SD* = 2.8; post: *M* = 25.8, *SD* = 3.3). There a significant interaction between intervention and diurnal cortisol slope, *F*(1, 15) = 4.83, *p* < 0.05, partial *η*^2^ = 0.25. Caregiving partners who received MBSR demonstrated an increase in diurnal cortisol rhythmicity (i.e., a steeper diurnal cortisol slope; pre: *M* = − 0.13, *SD* = 0.06; post: *M* = − 0.16, *SD* = 0.05), whereas those who received TAU demonstrated a decrease in diurnal cortisol rhythmicity (i.e., flatter diurnal cortisol slope; pre: *M* = − 0.14, *SD* = 0.08; post: *M* = − 0.10, *SD* = 0.05). No other relationships emerged as significant.

## Discussion

Importantly, no participants were lost to follow-up, suggesting MBSR was accepted by PD patients and caregiving-partner dyads and met a need early in the disease trajectory. Although exploratory rather than confirmatory, MBSR demonstrated differential effects for PD patients and their caregiving partners compared to control counterparts who received TAU. Patients in MBSR experienced a slight increase in overall perceived disease-specific distress and intrusive thoughts (one point) following the intervention, whereas patients who received TAU reported a large decrease in disease-specific distress (8 points) and intrusive thoughts (5 points) among patients in the TAU group. While these results contradict initial hypotheses, Baer et al. (2019) have noted that participation in mindfulness-based programs involves experiencing discomfort; unwanted thoughts, emotions, and sensations are expected to arise during practice. It is plausible mindfulness training increased introspective awareness and approach toward internal experience (Kabat-Zinn, 2003) leading patients to become more aware of internal experience, which may have involved distress and intrusive thoughts about their disease. Thus, it is possible patients were experiencing distress and intrusive thoughts prior to the intervention, but were less aware, and therefore, unable to report on such experiences. As posited by Dobkin and Zhao (2011), through increased awareness and contact with unpleasant internal experiences, MBSR patient participants may have subjectively felt worse before they learned to stay with difficult thoughts and emotions. It is also possible that potential benefits did not emerge until beyond the conclusion of the intervention and our assessment time point (Dobkin & Zhao, 2011). However, the possibility of harm should be acknowledged. Harm is defined by Baer et al. (2019) as a deterioration in the participants’ level of functioning as compared to baseline levels sustained as a result of the program. Because disease-specific distress was not assessed beyond completion of the intervention, it is unclear if these changes were sustained, and therefore, if the current findings qualify as harm. However, given that the increase in perceived distress/intrusive thoughts was small and other benefits were observed, harm is unlikely. Nonetheless, patients who received TAU and did not experience an additional intervention, which could have been perceived as burdensome, demonstrated a decrease in distress and intrusive thoughts. Future studies should assess distress longitudinally beyond completion of MBSR to elucidate whether benefits are realized after completing the program or if PD patients do not benefit psychologically from an MBSR intervention. This may be best addressed through use of an active control condition (another intervention) instead of TAU.

Patients who participated in the MBSR program also demonstrated a decrease in bedtime cortisol and IL-1beta compared to their control counterparts. These findings are particularly noteworthy given the growing evidence demonstrating significant alterations to the circadian system (Bordet et al., 2003; Breen et al., 2014) and increased inflammation (Alam et al., 2016) with the development and progression of PD. Circadian dysregulation, which promotes neuroinflammation, has been proposed as a risk factor for neurodegenerative disease (Leng et al., 2019). While it remains unclear whether circadian disruption is an early marker of neurodegeneration, a symptom of PD, or a result of dopaminergic treatment, because circadian dysregulation promotes neuroinflammation and neurodegeneration (Leng et al., 2019), non-pharmacologic interventions, such as MBSR, that evidence healthy shifts in these neurobiological systems is promising. It is plausible to hypothesize that improvements in circadian and immune function may halt or prevent the progress of the disease and alleviate symptoms, including daytime fatigue and sleep disruption without introducing additional pharmacologic-driven side effects. Alternatively, MBSR could have improved sleep and/or autonomic regulation (Brand et al., 2012) leading to observed improvements in biomarkers of circadian and immune function. Further investigation into these mechanisms is warranted.

MBSR-driven improvements were also observed among caregiving partners. Caregivers in the MBSR condition reported a significant increase in perceived social support and demonstrated improved circadian rhythmicity (i.e., steeper cortisol slopes). Evidence of improved social support is notable given that caregiving spouses of patients with PD have reported a progressive loss of social contacts as the disease worsens (Roland et al., 2010). Although qualitative data were not collected as a part of the current study, it is reasonable to hypothesize these reported improvements in perceived support may be a function of the group format of MBSR. Direct access and on-going interaction with other PD caregiving partners may have fostered a greater sense of social support compared to control counterparts. In addition to reported social benefits, improved circadian rhythmicity is of particular importance among caregivers given the growing evidence linking caregiving as a risk factor for poorer health (Schrag et al., 2006) and earlier mortality (Schulz & Beach, 1999). Because circadian systems are responsible for orchestrating a number of physiological processes that promote health and protect against disease, and dysregulated circadian function has been associated with a number of disease states (Gibson et al., 2009), an intervention that evidences healthy biological shifts in both PD patients and their caregiving partners who are at risk for stress and burden is clinically promising. Taken all together, these data are consistent with prior research demonstrating promise for MBSR as a biopsychosocial intervention for clinically ill populations, and extend that work to those affected by PD and caregiving partners.

Although caution in interpretation is warranted, results from the present study provide a few key points. First, the effects of MBSR may not always be immediate, or subjectively positive. Counter to our initial hypothesis, the increase in distress/intrusive thoughts reported by patients in the MBSR group is consistent with findings from other mindfulness-based intervention research. In a recent study conducted by Baer et al. (2021), two-thirds of a non-medically ill sample (*N* = 158) reported unpleasant experiences associated with mindfulness practice during an 8-week mindfulness course, but the majority (85–92%) rated these experiences as “not at all” or “somewhat” upsetting. Only 3–7% reported harm and only 2–7% demonstrated reliable deterioration on symptom questionnaires. While it is plausible an increase in reported distress/intrusive thoughts is a function of meditative practice (i.e., to increase awareness of internal experience), the reporting of non-significant and potentially harmful effects of mindfulness-based interventions is necessary and has been significantly lacking from the growing body of mindfulness research (Baer et al., 2019) and should not be overlooked in this sample. Second, we observed significant improvements in biomarkers of circadian function following MBSR in *both* PD patients *and* their caregiving partners in addition to a reduction in a biomarker of systemic inflammation (IL-1beta) in patients. This suggests salutary effects of MBSR may be experienced physiologically prior to, or in lieu of, psychological effects, although this should be explored further given the improvement in social support reported by caregiving partners. Finally, this study serves as an example of the intentional and feasible inclusion of PD caregiving partners in non-pharmacologic interventions, a relatively innovative approach that should continue to be explored and replicated.

### Limitations and Future Research

Despite evidence for improvements to health markers among patients and caregiving partners randomized to MBSR, limitations to the present study should be acknowledged. Due to the heterogeneity of the current sample, the generalizability of findings is limited to patients and caregiving partners early in the disease trajectory that identify as White, are primarily retired (53%), and are well resourced ($60,000–$80,000 median income). MBSR is a time-intensive intervention involving physical and cognitive practices rooted in Buddhist traditions that may not be feasible or accepted among patients and caregiving-partner dyads further in the disease trajectory and from other sociodemographic backgrounds. This remains to be tested and should be prioritized in future investigations. Given the relatively small sample size and pilot design, results should be interpreted with caution. The limited sample size did not allow for statistical examination of dyadic effects across the intervention. In other words, because the results of the current analysis do not account for the shared variance within a dyad, it is unclear if synergistic benefits occurred within a dyad due to co-participation. Future studies with greater statistical power should examine potential “crossover” effects of dyadic participation in MBSR to further clarify the benefits of simultaneous inclusion of caregiving partners. Lastly, we acknowledge the limitations associated with the use of TAU as a control condition (Kazdin, 2015), but remain encouraged by the promising results of the current investigation. Further examination into the effects of MBSR among PD patientcaregiver dyads in a more robust trial is warranted.

## Figures and Tables

**Table 1:** Sample characteristics (*N* = 36)

Variable	PD patients (*n* = 18)	Caregiving partners (*n* = 18^†^)
Age at study entry, mean (***SD***)	63.7 (7.0)	62.2 (10.4)
Gender		
Female	44.4%	50.0%
Male	55.6%	50.0%
Marital status		
Never married	5.6%	5.6%
Currently married	88.9%	88.9%
Divorced	0%	5.6%
Widowed	5.6%	0%
Race		
White	100%	100%
Education level		
High school	22.2%	33.3%
Associate/technical degree	27.8%	5.6%
Bachelor’s degree	33.3%	16.7%
Master’s degree	5.6%	5.6%
Doctoral/professional degree	11.1%	5.6%
Annual household income		
< $20,000	0%	5.6%
$20,000–39,999	11.1%	11.1%
$40,000–59,999	22.2%	16.7%
$60,000–79,999	11.1%	16.7%
$80,000–99,999	11.1%	5.6%
≥ $100,000	27.8%	11.1%
Employment status		
Full-time	33.3%	16.7%
Part-time	5.6%	16.7%
Homemaker or caregiver	5.6%	0%
Retired	50.0%	33.3%
Permanently disabled	5.6%	0%
Living situation		
Live alone	11.1%	11.1%
Live with significant other	83.3%	50%
Live with children/other relatives	5.6%	0%
Other	0%	5.6%

† Six caregiving partners did not provide demographic information on education, income, employment status, or living situation due to inadvertent changes in the demographic questionnaire part-way through the study

**Table 2:** MBSR intervention

Week	Attitudinal emphasis	Practice
1	Beginner’s mind	Body scan, 9-dot exercise, mindful eating
2	Non-judging	Body scan, sitting meditation, pleasant events, informal mindfulness of routine activity
3	Patience	Yoga, sitting meditation, unpleasant events, automatic pilot, mindful eating
4	Trust	Body scan, yoga, sitting meditation, mindful eating
5	Non-striving	Sitting meditation, body scan, yoga
6	Letting go	Sitting meditation, body scan, yoga, present moment focus, communications
All-day silent weekend retreat		
7	Acceptance	Sitting meditation, body scan, yoga, informal mindfulness
8	Maintenance	Resources

**Table 3:** *T*-test results of the randomization check at baseline

	Patient		*t*-test (*p*)	Caregiving partner		*t*-test (*p*)
	Control M (SD)	MBSR M (SD)		Control M (SD)	MBSR M (SD)	
Distress	16.3 (16.9)	12.3 (14.6)	.53 (.60)	9.6 (9.3)	14.1 (11.8)	− .88 (.39)
Social support	95.4 (14.0)	99.2 (14.1)	− .57 (.58)	105.0 (9.7)	100.9 (17.8)	.58 (.57)
Cortisol						
Overall mean	− 1.6 (.40)	− 1.7 (.18)	.69 (.50)	− 1.8 (.32)	− 1.7 (.35)	− 1.1 (.31)
Bedtime mean	− 2.7 (.70)	− 2.4 (.55)	− .99 (.34)	− 3.4 (.86)	− 3.0 (.56)	− 1.0 (.32)
Slope	− .10 (.03)	− .07 (.04)	− 1.8 (.09)	− .14 (.08)	− .13 (.06)	− .37 (.72)
Cytokines						
IL-6	.17 (.21)	.07 (.05)	1.3 (.25)	.25 (.28)	.46 (1.1)	− .49 (.63)
TNF-alpha	3.6 (2.2)	4.5 (6.1)	− .38 (.71)	4.4 (3.2)	4.2 (1.2)	.15 (.89)
IL-1beta	1.2 (1.3)	1.6 (1.2)	− .54 (.60)	1.9 (.88)	2.9 (2.8)	− .96 (.36)

## Data Availability

Full access to the data is available upon reasonable request. Researchers who wish to access the data should contact the corresponding author.
